# Assessment of Knowledge, Clinical Experience, and Clinical Decision-Making Regarding Coronectomy of Mandibular Third Molars Among General Dentists and Oral and Maxillofacial Surgeons: A Cross-Sectional Survey

**DOI:** 10.3390/healthcare14172868

**Published:** 2026-09-07

**Authors:** Alperen Kırkpunar, İnci Rana Karaca

**Affiliations:** Department of Oral and Maxillofacial Surgery, Faculty of Dentistry, Gazi University, 06490 Ankara, Turkey; incirana@gazi.edu.tr

**Keywords:** cross-sectional studies, dentists, knowledge, mandibular nerve, molar, third, oral and maxillofacial surgeons, tooth, impacted

## Abstract

**Background/Objectives:** Coronectomy is a nerve-preserving alternative for mandibular third molars at high risk of inferior alveolar nerve injury. However, coronectomy-related knowledge, clinical experience, treatment preferences, and self-reported decision-making confidence may differ between general dentists (GD group) and oral and maxillofacial surgeons (OMFS group). This study aimed to compare these factors and identify factors associated with coronectomy-related knowledge. **Methods:** This cross-sectional survey was conducted between June 2024 and January 2026 among 300 dentists in Turkey. Participants were divided into the OMFS group (n = 153) and GD group (n = 147) and recruited using convenience sampling. A 34-item questionnaire assessed objective and self-perceived knowledge, attitudes, clinical experience, treatment preferences, and self-reported decision-making confidence. Total knowledge scores were calculated from correct responses. Non-parametric tests, chi-square tests, Spearman’s correlation, item-level analysis, and multivariable linear regression were performed. **Results:** The median knowledge score was 39 (IQR: 9) out of a maximum possible score of 47. The OMFS group had higher knowledge scores, greater self-reported decision-making confidence, and more coronectomy experience than the GD group. Self-perceived knowledge showed a moderate positive correlation with total knowledge score (ρ = 0.414, *p* < 0.001). Item-level analysis identified lower correct response rates for radiographic risk markers, contraindications, and coronectomy-related complications. Professional group, prior awareness of coronectomy, and preference for three-dimensional imaging were independently associated with total knowledge score; however, the multivariable model explained only a modest proportion of the variance (adjusted R^2^ = 0.169). **Conclusions:** Coronectomy-related awareness was high; however, knowledge, experience, and self-reported decision-making confidence differed between the groups. Awareness alone may be insufficient for self-reported confidence in decision-making. These finding s may help identify areas for targeted continuing education and structured clinical guidance, particularly in radiographic risk assessment, case selection, and referral decisions.

## 1. Introduction

The surgical removal of impacted mandibular third molars is one of the most commonly performed procedures in oral and maxillofacial surgery and may be associated with intraoperative and postoperative complications [1,2]. Recent evidence has also shown that mandibular third molar extraction carries a higher complication risk than maxillary third molar extraction [3], emphasizing the clinical relevance of complications associated with this frequently performed procedure [4]. Among these, inferior alveolar nerve (IAN) injury is one of the most clinically significant, with a reported incidence ranging from 0.3% to 8.4% and, in some cases, resulting in permanent sensory deficits [5]. To reduce the risk of IAN injury, the coronectomy technique was introduced as an alternative to the surgical removal of mandibular third molars with a high risk of nerve injury. In this procedure, the crown of the tooth is removed while the roots are intentionally retained within the alveolar bone to preserve the IAN [6]. Coronectomy is most commonly performed on mandibular third molars; however, it has also been reported in rare cases in deeply impacted second molars and, exceptionally, in first molars [7].

Since it was first described by Ecuyer and Debien [8] in 1984, the coronectomy technique has been approached with caution by clinicians [9]. The limited availability of long-term outcome data and concerns regarding potential complications remain among the main factors limiting the routine adoption of coronectomy in clinical practice [10]. Coronectomy was reported to reduce the incidence of paresthesia, while the rates of other complications appear comparable to those associated with conventional surgical extraction in most studies [11]. Although the evidence base for coronectomy has expanded, variations in case selection, follow-up duration, and clinician acceptance continue to influence its implementation in practice. These findings suggest that coronectomy may reduce the risk of IAN injury compared with conventional surgical extraction of mandibular third molars [12,13,14]. However, coronectomy is also associated with procedure-specific outcomes. In a systematic review, failed coronectomy was reported in 2.3–38.3% of cases, root migration in 13.2–85.29%, root exposure in 0–1.3%, and reoperation in 0–4.9% [15]. Although root migration is frequently observed following coronectomy, it has been reported that, in most cases, migration occurs away from the IAN, and only a small proportion of patients require secondary surgical intervention [7]. In rare cases, continued root migration may result in eruption into the oral cavity, a process that has been reported to occur over several years. The decision to remove retained roots should be based on clinical symptoms rather than the presence of retained roots alone [11,16].

The coronectomy technique has gained increasing acceptance in recent clinical reports and was included as a viable surgical option in the 2012 American Association of Oral and Maxillofacial Surgeons (AAOMS) clinical practice guidelines for the management of third molars [17,18].

Despite these positive findings, a study conducted by Crameri et al. [19] among maxillofacial surgeons in Switzerland to evaluate their attitudes toward coronectomy reported that 51.6% of participants considered the technique to be unreliable. In a study conducted by Alqhtani et al. [20], 38.9% of dentists did not consider the coronectomy technique to be reliable.

Although previous surveys have explored clinicians’ awareness, attitudes, and perceptions regarding coronectomy, comparatively little attention has been given to objectively assessed coronectomy-related knowledge and its relationship with clinical experience, treatment preferences, and self-reported decision-making confidence. In addition, direct comparisons between general dentists and clinicians with oral and maxillofacial surgery training across these domains remain limited. Therefore, the present study aimed to assess and compare coronectomy-related knowledge, clinical experience, treatment preferences, and self-reported decision-making confidence between oral and maxillofacial surgeons (OMFS group) and general dentists (GD group), and to identify factors independently associated with coronectomy-related knowledge.

The null hypothesis was that there would be no significant difference in total coronectomy-related knowledge scores between the OMFS and GD groups.

## 2. Materials and Methods

This cross-sectional survey was conducted between June 2024 and January 2026 using face-to-face questionnaires. The study protocol was approved by the Ethics Committee of Gazi University (approval date: 14 May 2024; meeting no.: 09; approval number: 2024-804). Written informed consent was obtained from all participants before questionnaire completion and was documented by each participant’s signature on an informed consent form. To reduce potential response bias, participants were informed that participation was voluntary, responses would remain anonymous, and their answers would not have any negative consequences. This study was reported in accordance with the Strengthening the Reporting of Observational Studies in Epidemiology (STROBE) checklist for cross-sectional studies. The completed STROBE checklist is provided as Supplementary File S3.

### 2.1. Study Design and Participants

The study population consisted of dentists practicing in Turkey across public hospitals, private dental clinics, and university hospitals. Participants were divided into two groups according to professional background. The oral and maxillofacial surgeons group (OMFS group) included oral and maxillofacial surgery specialists and oral and maxillofacial surgery residents. The general dentists group (GD group) included dentists without oral and maxillofacial surgery specialty training. These two groups constituted the main comparison groups of the study. Residents and specialists were combined within the OMFS group because the primary grouping was based on exposure to specialty training in oral and maxillofacial surgery rather than specialist status alone. Both residents and specialists had received or were receiving OMFS-specific training and were therefore considered distinct from dentists without such specialty training. Workplace sector was recorded as a single-response variable (public or private), and these categories were treated as mutually exclusive for the purposes of the survey. Specific institution type (public hospital, private dental clinic, or university hospital) was not recorded as a separate analytical variable; workplace was recorded only as public or private sector.

Participants were recruited using convenience sampling from public hospitals, private dental clinics, and university hospitals. Eligible dentists were approached face-to-face by the researchers during the study period in institutions and clinical settings accessible to the research team. Dentists who were actively practicing at the time of the study and agreed to participate were included. Participation was voluntary.

As recruitment was based on face-to-face convenience sampling rather than a predefined sampling frame or formal invitation list, a response rate could not be calculated. Convenience sampling was used because the target population included dentists working in different healthcare settings and because no centralized sampling frame stratified by coronectomy-related clinical experience was available. No geographic quota or regional stratification was applied; therefore, the sample should be interpreted as a convenience sample of dentists practicing in Turkey rather than as a nationally representative sample.

Since the questionnaires were administered face-to-face, participants were asked to complete all applicable items before submission, and no incomplete questionnaires were included in the dataset. The minimum required sample size was calculated a priori using G*Power version 3.1.9.2 for a chi-square test using contingency tables. The calculation was based on an effect size of w = 0.174, estimated from published data of a previous study investigating coronectomy-related knowledge and attitudes among dentists [20], with α = 0.05, a statistical power of 80%, and 1 degree of freedom. The resulting minimum required sample size was 259 participants (actual power = 0.801). Because the calculation was based on a chi-square contingency-table test, an allocation ratio, one- versus two-sided specification, and number of regression predictors were not applicable. A total of 300 participants were included in the present study.

### 2.2. Questionnaire Design

A structured questionnaire developed by the researchers was used as the data collection tool. The questionnaire was prepared based on a review of previously published studies and was designed to address the objectives of the present study.

The questionnaire was evaluated by a panel of six experts in oral and maxillofacial surgery, oral radiology, and clinical dentistry. The CVR values ranged from 0.67 to 1.00 across the 34 items. The item-level CVI values for both relevance and clarity were 1.00 for all items, resulting in an S-CVI/Ave of 1.00. Based on expert feedback, no item was removed; however, minor wording revisions were made to improve clarity.

After content validity assessment, the revised questionnaire was pilot tested among 20 dentists, including 10 general dentists and 10 oral and maxillofacial surgeons, who were not included in the final study sample. The pilot test evaluated the clarity of the items, appropriateness of the response options, and questionnaire completion time. The mean completion time was approximately 11 min. No major structural changes were made after pilot testing; however, items were grouped into thematic sections, instructions for multiple-response questions were clarified, and minor wording revisions were made. 

The final questionnaire consisted of 34 items assessing demographic and professional characteristics, clinical experience with impacted mandibular third molar surgery, objective knowledge of impacted mandibular third molar surgery, inferior alveolar nerve injury, radiographic risk markers, and coronectomy, as well as self-perceived knowledge, attitudes toward coronectomy, treatment preferences, self-reported decision-making confidence, and coronectomy-related clinical experience. Knowledge items included multiple-choice, multiple-response, and “Yes/No/Do not know” questions. Consistent with previous questionnaire-based dental studies in which correct responses to knowledge-related items were scored and summed to generate an overall knowledge score, each correct response was assigned one point [21,22,23]. Incorrect responses, unselected correct options, and “Do not know” responses were scored as zero. For multiple-response questions, each correct option was evaluated separately. Specific items (Q21–Q23) were structured as “Yes/No/Do not know” questions, in which “Yes” responses were considered correct and assigned one point, whereas “No” and “Do not know” responses were scored as zero. The total knowledge score comprised Q10 (9 scorable response options), Q12 (7 options), Q14 (6 options), Q16 (7 options), Q21–Q23 (3 items), Q25 (7 options), and Q27 (8 options), yielding a possible total score ranging from 0 to 47. An evidence-based answer key specifying the scored response options and their supporting references is provided in Supplementary File S4.

Self-perceived knowledge was assessed using a five-point Likert scale. The self-perceived knowledge score was calculated as the mean of four Likert-scale items, with higher scores indicating greater self-perceived knowledge. Open-ended questions were included to allow participants to provide additional details regarding their clinical experiences. The questionnaire used in the study is provided as Supplementary File S1.

### 2.3. Study Variables

The primary outcome variable was the total coronectomy-related knowledge score, calculated as the sum of correct responses to the objective knowledge items. The main independent variable was professional group. Participants were categorized as OMFS group or GD group. The OMFS group included oral and maxillofacial surgery specialists and residents, whereas the GD group included dentists without oral and maxillofacial surgery specialty training. Secondary variables included prior awareness of coronectomy, self-perceived knowledge, attitudes toward coronectomy, self-reported decision-making confidence, clinical experience with coronectomy, self-reported complications, sources of coronectomy-related knowledge, and treatment preferences for mandibular third molars in close proximity to the inferior alveolar nerve. Other variables considered in the analyses included gender, years of professional experience, workplace sector, postgraduate continuing education, and preference for three-dimensional imaging.

### 2.4. Statistical Analysis

Statistical analyses were performed using the Statistical Package for the Social Sciences (SPSS) for Windows, version 27.0 (SPSS Inc., Chicago, IL, USA). Descriptive statistics were presented as frequencies and percentages for categorical variables. Continuous variables were assessed for normality using the Shapiro–Wilk test. Normally distributed continuous variables were summarized as means ± standard deviations, whereas non-normally distributed continuous variables, including the total knowledge score, were summarized as medians, interquartile ranges (IQR), and minimum–maximum values. Because the total knowledge score was not normally distributed, non-parametric tests were used for group comparisons. Comparisons between two independent groups were performed using the Mann–Whitney U test, while comparisons among three or more independent groups were conducted using the Kruskal–Wallis test. When a statistically significant difference was detected, Bonferroni-adjusted post hoc analysis was performed to identify the source of the difference. Relationships between continuous variables were evaluated using Spearman’s rank correlation coefficient. Associations between categorical variables were analyzed using the chi-square test. Fisher’s exact test or the Fisher–Freeman–Halton test was used when expected cell counts were insufficient.

For the item-level analysis, the proportion of correct responses was calculated separately for each knowledge item. For multiple-response questions, each option was evaluated as a separate item, and percentages were calculated using the total number of participants as the denominator. Responses to open-ended questions were analyzed using qualitative content analysis and categorized into thematic groups based on recurring patterns. For Question 34, only non-blank responses from participants who reported previous coronectomy experience were included in the complication analysis. Blank responses were treated as missing/non-response and were not interpreted as indicating the absence of complications. The knowledge scale demonstrated high internal consistency (Cronbach’s alpha = 0.852), and the self-perceived knowledge items showed excellent internal consistency (Cronbach’s alpha = 0.927). To assess whether total knowledge score was associated with treatment preferences independently of professional group, separate binary logistic regression analyses were performed for extraction, coronectomy, and referral. Each treatment preference was entered as a binary dependent variable (selected vs. not selected), with total knowledge score and professional group included as independent variables. Odds ratios (ORs), 95% confidence intervals (CIs), and *p*-values were reported.

Multivariable linear regression analysis was performed to identify factors independently associated with total knowledge score. The independent variables included professional group, years of professional experience, postgraduate continuing education, prior awareness of coronectomy, and preference for three-dimensional imaging. These variables were selected a priori as background or exposure-related factors. Variables reflecting self-reported decision-making confidence, treatment preferences, or coronectomy experience were not included in the main regression model because they were considered conceptually related to, or potentially downstream of, coronectomy-related knowledge rather than baseline explanatory variables.

Before interpretation of the regression model, linear regression assumptions were evaluated. Independence of errors was examined using the Durbin–Watson statistic, normality of residuals was evaluated using residual plots and normal probability plots, and multicollinearity was assessed using tolerance and variance inflation factor (VIF) values. Regression coefficients, standard errors, standardized beta coefficients, 95% confidence intervals, *p*-values, and VIF values were reported. A *p*-value < 0.05 was considered statistically significant for all analyses.

## 3. Results

### 3.1. Participant Characteristics

A total of 300 completed questionnaires were included in the final analysis, comprising 153 (51.0%) participants in the OMFS group and 147 (49.0%) in the GD group. Gender distribution differed significantly between the professional groups (*p* < 0.001), with a higher proportion of male participants in the OMFS group (64.1%) and a higher proportion of female participants in the GD group (66.7%). Workplace sector also differed significantly between the groups (*p* = 0.029), with a higher proportion of participants working in the public sector in the OMFS group than in the GD group (88.2% vs. 78.9%). No significant between-group differences were observed in professional experience (*p* = 0.434) or postgraduate continuing education (*p* = 0.789). Detailed demographic and professional characteristics are presented in Table 1.

### 3.2. Comparison of Knowledge Scores Between Professional Groups

Table 2A presents the comparison of total knowledge scores between the OMFS and GD groups. The OMFS group had significantly higher total knowledge scores than the GD group (median: 41 vs. 36; *p* < 0.001), with a moderate effect size (r = 0.377).

### 3.3. Knowledge Scores and Related Factors

Table 2B presents total knowledge scores according to selected variables. The median total knowledge score was 39 (IQR: 9). Knowledge scores differed significantly according to perceived reliability of coronectomy. Bonferroni-adjusted post hoc comparisons showed that participants who responded “I do not know” had significantly lower knowledge scores than those who responded “Yes” (adjusted *p* < 0.001) and “No” (adjusted *p* < 0.001), whereas no significant difference was observed between the “Yes” and “No” groups (adjusted *p* = 0.345). Knowledge scores also differed significantly according to coronectomy experience. Participants with no coronectomy experience had significantly lower knowledge scores than those with fewer than five cases (adjusted *p* = 0.017) and those with five or more cases (adjusted *p* = 0.001), whereas the latter two groups did not differ significantly (adjusted *p* = 1.000). No significant differences were observed according to years of professional experience, workplace sector, or interest in continuing education on coronectomy (all *p* > 0.05).

### 3.4. Item-Level Analysis of Knowledge Questions

Item-level analysis of the knowledge questions is presented in Supplementary File S2. Correct response rates varied across knowledge domains. High correct response rates were observed for root resorption of the adjacent tooth as an indication for extraction (98.3%), infection as a complication of extraction (97.7%), third molar surgery as a procedure associated with inferior alveolar nerve injury (99.7%), root retention with crown removal in coronectomy (95.0%), and postoperative pain as a complication of coronectomy (94.7%). In contrast, lower correct response rates were found for periodontitis as an indication for extraction (66.7%), narrowing of the root (38.7%) and narrowing of the canal (54.0%) as radiographic markers, horizontal positioning along the course of the inferior alveolar nerve as a contraindication for coronectomy (52.3%), and nerve injury as a potential complication of coronectomy (45.3%).

### 3.5. Comparison of Selected Coronectomy-Related Variables Between Professional Groups

Table 3 presents the comparison of selected coronectomy-related variables between the OMFS and GD groups. The OMFS group considered coronectomy reliable more frequently than the GD group (75.8% vs. 53.1%), whereas uncertainty regarding the reliability of coronectomy was substantially more common among the GD group (40.1% vs. 10.5%) (*p* < 0.001). Similarly, a significantly higher proportion of participants in the OMFS group reported confidence in deciding between coronectomy and extraction than in the GD group (81.0% vs. 17.0%, *p* < 0.001). Previous clinical experience with coronectomy was also significantly more common in the OMFS group, whereas lack of experience was markedly higher in the GD group (*p* < 0.001). In addition, prior awareness of coronectomy was significantly more frequent in the OMFS group than in the GD group (99.3% vs. 87.1%; *p* < 0.001). However, no significant differences were found between the groups regarding interest in continuing education on coronectomy (*p* = 0.145) or preference for three-dimensional imaging (*p* = 0.081).

### 3.6. Self-Perceived Knowledge Score

Participants’ self-perceived knowledge was evaluated using four five-point Likert-scale items, and a mean self-perceived knowledge score was calculated (Table 4). Among these items, the highest mean score was observed for knowledge of third molar surgery complications, whereas the lowest was observed for knowledge of coronectomy complications. Self-perceived knowledge showed a moderate positive correlation with total knowledge score (ρ = 0.414, *p* < 0.001). In addition, the OMFS group had significantly higher self-perceived knowledge scores than the GD group (*p* < 0.001).

### 3.7. Analysis of Self-Reported Complications

Among the 109 participants who reported previous coronectomy experience, 52 (47.7%) provided a non-blank response to Question 34, whereas 57 (52.3%) left the question blank. Blank responses were treated as missing/non-response and were not interpreted as indicating the absence of complications. The 52 respondents provided a total of 135 complication reports. As shown in Table 5A, the most frequently reported complications were infection, postoperative pain, and root eruption or migration.

### 3.8. Sources of Knowledge About Coronectomy

Sources of coronectomy-related knowledge are presented in Table 5B. Undergraduate education was the most frequently reported source, followed by specialty training and scientific literature/textbooks. Clinical experience, colleagues/peer learning, and courses/seminars/social media were reported less frequently.

### 3.9. Treatment Preferences According to Professional Group and Knowledge Score

Participants were allowed to select more than one treatment option. Table 6 shows the association between professional group and treatment preferences for mandibular third molars in close proximity to the inferior alveolar nerve. The OMFS group selected extraction and coronectomy significantly more frequently than the GD group, whereas the GD group was significantly more likely to prefer referral to a specialist (all *p* < 0.001). No significant association was observed between professional group and the selection of “wait-and-see” or orthodontic extrusion followed by extraction (*p* > 0.05).

Total knowledge scores according to treatment preferences are presented in Table 7. Participants who selected complete extraction had significantly higher knowledge scores than those who did not select this option (median: 41 vs. 37; *p* < 0.001). Similarly, participants who selected coronectomy had higher knowledge scores than those who did not select coronectomy (median: 40 vs. 36; *p* < 0.001). In contrast, participants who selected referral to a specialist had lower knowledge scores than those who did not select referral (median: 36 vs. 40; *p* < 0.001). No significant differences were observed according to selection of the wait-and-see option or orthodontic extrusion followed by extraction (*p* > 0.05).

To further assess whether the total knowledge score was independently associated with treatment preferences after accounting for professional group, separate binary logistic regression analyses were performed. After adjustment for professional group, higher total knowledge score remained significantly associated with greater odds of selecting extraction (OR = 1.066, 95% CI: 1.015–1.120, *p* = 0.011) and coronectomy (OR = 1.061, 95% CI: 1.008–1.116, *p* = 0.023). In contrast, the association between total knowledge score and referral to a specialist was no longer statistically significant after adjustment for professional group (OR = 0.951, 95% CI: 0.902–1.002, *p* = 0.060).

### 3.10. Multivariable Linear Regression Analysis

Multivariable linear regression analysis was performed to identify factors independently associated with total knowledge score (Table 8). The overall regression model was statistically significant (F = 11.10, *p* < 0.001) and explained 16.9% of the variance in total knowledge score (adjusted R^2^ = 0.169; R^2^ = 0.185).

After adjustment for the variables included in the model, professional background, prior awareness of coronectomy, and preference for three-dimensional imaging remained independently associated with total knowledge score. Compared with oral and maxillofacial surgeons, general dentists had significantly lower knowledge scores (B = −3.777, *p* < 0.001). Participants who had previously heard of coronectomy had significantly higher knowledge scores than those who had not (B = 2.983, *p* = 0.021). Likewise, participants who preferred three-dimensional imaging for high-risk mandibular third molars had significantly higher knowledge scores than those who did not (B = 2.639, *p* = 0.006). Years of professional experience and postgraduate continuing education were not independently associated with total knowledge score (all *p* > 0.05).

## 4. Discussion

Coronectomy has been proposed as a nerve-preserving alternative for mandibular third molars at high risk of inferior alveolar nerve injury [6]. Although increasing evidence supports its effectiveness, its adoption in clinical practice remains variable [12,13,14,15,19,20,24,25]. The present study showed that coronectomy-related knowledge, clinical experience, treatment preferences, and self-reported decision-making confidence differed between the OMFS and GD groups. The OMFS group had higher knowledge scores, greater clinical experience, and higher self-reported decision-making confidence than the GD group. Accordingly, the null hypothesis was rejected, as total coronectomy-related knowledge scores were significantly higher in the OMFS group than in the GD group. In addition, item-level variability in knowledge and differences in treatment preferences according to knowledge score suggest a discrepancy between awareness and clinically applicable decision-making. Because of the cross-sectional design, these findings should be interpreted as associations rather than causal relationships.

Several studies have reported that coronectomy is not routinely adopted in clinical practice, despite growing evidence supporting its effectiveness in selected high-risk mandibular third molar cases. In a UK survey, some oral and maxillofacial surgeons reported not performing coronectomy because of concerns about insufficient evidence, the possibility of secondary surgery, and postoperative complications associated with the technique [26]. In line with this, Crameri et al. reported in a Swiss survey that 51.6% of participants did not consider coronectomy to be a reliable technique [19]. This hesitation may partly reflect the broad range of complications associated with third molar surgery in general, with reported complication rates ranging from 4.6% to 30.9% [4,27]. This hesitation may also be influenced by coronectomy-specific concerns, such as root migration and the potential need for secondary intervention. Nevertheless, coronectomy appears to be associated with a lower risk of inferior alveolar nerve injury than complete extraction, while rates of other postoperative complications, including pain, infection, delayed healing, and alveolar osteitis, are generally considered comparable [10,17]. In the present study, 64.7% of participants considered coronectomy reliable, whereas one quarter reported uncertainty. This finding suggests that, even when overall perception is relatively favorable, uncertainty regarding the long-term management and clinical predictability of coronectomy may remain a barrier to confident clinical adoption.

The OMFS group had higher knowledge scores, greater self-reported decision-making confidence, and more clinical experience with coronectomy than the GD group. This pattern is clinically expected, as OMFS training and practice involve greater exposure to surgical case selection, radiographic risk assessment, operative management, and procedure-specific complications. Accordingly, these between-group differences should not be interpreted as evidence of an educational deficiency among general dentists, whose clinical responsibilities and exposure to high-risk mandibular third molar surgery differ from those of OMFS clinicians. Rather, the observed differences may reflect a combination of specialty-specific training, clinical exposure, the types and complexity of cases encountered, workplace-related factors, and other individual or institutional characteristics, rather than professional education alone. Overall, the present findings are broadly consistent with international survey data, although direct comparisons should be made cautiously because previous studies differed in their study populations and methodologies. In Saudi Arabia, Alqhtani et al. [20] reported that 71.9% of dentists were aware of coronectomy, 61.1% considered the procedure reliable, and 40.6% reported confidence in deciding between coronectomy and extraction, suggesting a similar discrepancy between awareness and decision-making confidence. Beaumont et al. [28] likewise reported differences between general dental practitioners and specialist clinicians in coronectomy-related decision-making. Surveys from Switzerland and India also demonstrated variability in coronectomy-related perceptions among clinicians with different professional backgrounds and levels of clinical exposure [19,25]. Differences across countries may reflect variation in professional training, clinical exposure, imaging practices, referral patterns, and local approaches to coronectomy; however, differences in participant composition and study methodology should also be considered when interpreting these comparisons.

The present findings also indicate that prior awareness of coronectomy does not necessarily translate into clinical experience or confidence in decision-making. Although most participants had heard of coronectomy, clinical experience with the procedure was limited, particularly in the GD group. Higher knowledge scores were associated with greater self-reported confidence in choosing between coronectomy and complete extraction, previous coronectomy experience, and the selection of extraction or coronectomy. In the unadjusted analyses, participants who selected complete extraction or coronectomy had higher knowledge scores, whereas those who selected referral to a specialist had lower knowledge scores. However, after adjustment for professional group, total knowledge score remained significantly associated with the selection of extraction and coronectomy, whereas the association between knowledge score and referral to a specialist was no longer statistically significant. Therefore, the unadjusted association between lower knowledge scores and referral should not be interpreted as an independent relationship. Referral to a specialist may represent an appropriate clinical management strategy, particularly for clinicians who do not routinely perform high-risk mandibular third molar surgery. These findings suggest that both professional background and coronectomy-related knowledge may contribute to differences in treatment preferences. Similar discrepancies between awareness, confidence, and clinical implementation have also been reported in previous survey-based studies [20,25].

Concerns regarding postoperative complications may also contribute to uncertainty in clinical decision-making. In the present study, the most frequently self-reported complications were infection, pain, and root eruption or migration. These findings are consistent with previous reports showing that most complications after coronectomy are comparable to those after conventional third molar surgery, although root migration is a more characteristic outcome of coronectomy. Although coronectomy is associated with a lower risk of inferior alveolar nerve injury than complete extraction, concerns about postoperative complications and root-related outcomes may still limit its routine clinical adoption [17].

The distribution of knowledge sources indicates that coronectomy-related awareness was most frequently reported as originating from undergraduate education. However, the present cross-sectional data do not allow differences in clinically applicable knowledge or self-reported decision-making confidence to be attributed specifically to educational background, as these outcomes may also be influenced by clinical exposure, caseload, age, workplace characteristics, and institutional factors. This interpretation is supported by the item-level findings, which showed lower correct response rates for selected radiographic risk markers, contraindications, and coronectomy-related complications. Therefore, future educational efforts should move beyond general awareness and focus on radiographic risk assessment, patient selection, contraindications, management of retained roots, postoperative follow-up, and clear referral pathways for high-risk cases involving suspected inferior alveolar nerve proximity [20,25].

The treatment preferences observed in the present study can be interpreted alongside the findings of Crameri et al. [19], who used a similar multiple-response question for mandibular third molars at high risk of inferior alveolar nerve injury. In their study, operative removal was selected by 93.5% of respondents, leaving the tooth in situ or adopting a wait-and-see approach by 79.4%, coronectomy by 40.6%, referral by 12.9%, and orthodontic extrusion by 2.6%. In the present study, oral and maxillofacial surgeons selected complete extraction and wait-and-see options at comparable but somewhat lower rates, whereas their selection of coronectomy was considerably higher. By contrast, the GD group in the present study selected referral to a specialist more frequently than the Swiss specialist cohort. These differences may reflect variation in professional background, clinical responsibility, and confidence in managing high-risk third molar cases. Knowledge score was also associated with treatment preference in the unadjusted analyses. However, after adjustment for professional group, higher knowledge scores remained significantly associated with the selection of complete extraction and coronectomy, whereas the association with referral to a specialist was no longer statistically significant. This finding suggests that professional background may account, at least in part, for the observed unadjusted association between lower knowledge scores and referral. Referral should not be interpreted as a less appropriate management option, particularly when inferior alveolar nerve proximity is suspected or when the clinician lacks sufficient experience in case selection or surgical management. 

Multivariable regression analysis showed that professional group, prior awareness of coronectomy, and preference for three-dimensional imaging were independently associated with total knowledge score after adjustment for selected background and exposure-related variables. The GD group had significantly lower knowledge scores than the OMFS group, which may reflect differences in specialty-specific training, exposure to high-risk mandibular third molar cases, and familiarity with coronectomy-related decision-making. The association between prior awareness and higher knowledge scores is clinically plausible, as awareness may represent initial exposure to the concept of coronectomy. However, previous survey-based studies indicate that awareness alone does not necessarily translate into clinical experience or self-reported decision-making confidence. For example, Alqhtani et al. [20] reported that although most dentists were aware of coronectomy, fewer had performed or recommended the procedure, and less than half reported confidence in deciding between coronectomy and complete extraction.

The association between preference for three-dimensional imaging and higher knowledge scores may reflect greater familiarity with radiographic risk assessment in high-risk mandibular third molar cases. However, this finding should not be interpreted as supporting routine CBCT use for all mandibular third molars. Robbins et al. [29] reported that adding CBCT to panoramic imaging does not routinely reduce the incidence of inferior alveolar nerve injury during mandibular third molar removal. Therefore, clinicians who prefer three-dimensional imaging in high-risk situations may be more attentive to anatomical risk assessment and case selection, rather than three-dimensional imaging preference itself indicating better clinical outcomes. Nevertheless, the model explained only 16.9% of the variance in total knowledge score, indicating that additional unmeasured factors, such as undergraduate curriculum differences, institutional clinical volume, exposure to high-risk cases, mentorship, referral patterns, and case-based continuing education, may also contribute to coronectomy-related knowledge. Accordingly, these regression findings should be interpreted as associations rather than causal explanations.

From a clinical perspective, the present findings highlight the importance of structured decision-making when managing mandibular third molars in close proximity to the inferior alveolar nerve. Differences in coronectomy-related knowledge may have practical implications for radiographic risk assessment, recognition of indications and contraindications, patient counselling, treatment selection, and referral decisions. Coronectomy may be considered when the risk of inferior alveolar nerve injury is high and no contraindications are present, whereas complete extraction may remain appropriate when the risk of nerve injury is low or when coronectomy is contraindicated. Conversely, uncertainty regarding radiographic risk markers, case selection, or procedure-specific complications may contribute to avoidance of coronectomy or selection of complete extraction despite high-risk features. For clinicians with limited coronectomy experience or uncertainty in radiographic interpretation and case selection, referral to a specialist should be regarded as a prudent clinical strategy rather than an unfavorable decision. Clear referral pathways and case-based clinical guidance may help reduce uncertainty and support more appropriate treatment planning.

The present study has several limitations. First, its cross-sectional design precludes causal inference. Therefore, the observed associations between total knowledge score and coronectomy-related perceptions, clinical experience, treatment preferences, or self-reported decision-making confidence should not be interpreted as indicating directionality. It remains unclear whether greater coronectomy-related knowledge contributes to higher confidence and clinical use, or whether greater clinical exposure increases knowledge over time.

Second, participants were recruited using convenience sampling, which may have introduced selection bias and limits the generalizability of the findings to all dentists in Turkey. Dentists who were more accessible, worked in institutions with greater exposure to oral surgery, or had a stronger interest in mandibular third molar surgery may have been more likely to participate. In addition, the sample included a high proportion of dentists working in the public sector, and regional distribution was not analyzed; therefore, the findings should not be interpreted as nationally representative.

Third, the study relied on self-reported questionnaire data, which may have introduced recall bias, response bias, and social desirability bias. Participants’ reported confidence in deciding between coronectomy and complete extraction may not fully reflect their clinical performance in real-life scenarios. Similarly, reported clinical experience and complications were not verified through clinical records. In addition, Question 34 did not include an explicit “no complications” response option; therefore, blank responses were treated as missing/non-response and excluded from the complication analysis. Consequently, the complication findings were based only on participants who provided non-blank responses and may not fully represent the experiences of all participants who had previously performed coronectomy.

Fourth, although the OMFS group included dentists with completed or ongoing specialty training, residents and specialists were analyzed together. Resident status, residency year, specialist seniority, and institutional surgical case volume were not recorded as separate variables. Therefore, potential differences related to training level, seniority, or clinical exposure within the OMFS group could not be evaluated.

Finally, the multivariable linear regression model explained only a limited proportion of the variance in total knowledge score. This suggests that additional unmeasured factors, such as undergraduate curriculum differences, institutional clinical volume, exposure to high-risk mandibular third molar cases, access to mentorship, local referral patterns, CBCT availability and use, and participation in case-based continuing education, may also contribute to coronectomy-related knowledge. Therefore, the regression findings should be interpreted as adjusted associations rather than as evidence of causal relationships or a comprehensive explanatory model. Future studies should use multicenter designs with probability-based or stratified sampling, include geographic and institutional representation, assess resident year and specialist seniority, and evaluate the effectiveness of case-based educational interventions using pre- and post-training designs.

## 5. Conclusions

The present findings suggest that awareness of coronectomy alone may not necessarily correspond to clinically applicable knowledge, self-reported decision-making confidence, or procedure-specific clinical experience. Differences in professional background, clinical exposure, and knowledge level were associated with how participants reported coronectomy-related decision-making. The variability in item-level knowledge, limited clinical experience, and differences in treatment preferences according to knowledge score indicate a potential discrepancy between awareness, knowledge, and self-reported decision-making confidence. Because of the cross-sectional design, these findings should be interpreted as associations rather than causal relationships. Targeted education, case-based training, and structured clinical guidance may help address areas of uncertainty in radiographic risk assessment, case selection, referral decisions, and evidence-based use of coronectomy; however, longitudinal or interventional studies are needed to evaluate their effectiveness.

## Figures and Tables

**Table 1:** Demographic and Professional Characteristics of Participants Overall and by Professional Group.

Variable	Total (n = 300)	OMFS (n = 153)	GD (n = 147)	*p*-Value
**Gender**				<0.001 *
Female	153 (51.0)	55 (35.9)	98 (66.7)	
Male	147 (49.0)	98 (64.1)	49 (33.3)	
**Professional experience**				0.434
<5 years	189 (63.0)	91 (59.5)	98 (66.7)	
5–10 years	82 (27.3)	46 (30.1)	36 (24.5)	
>10 years	29 (9.7)	16 (10.5)	13 (8.8)	
**Workplace sector**				0.029 *
Public sector	251 (83.7)	135 (88.2)	116 (78.9)	
Private sector	49 (16.3)	18 (11.8)	31 (21.1)	
**Postgraduate continuing education**				0.789
No	112 (37.3)	56 (36.6)	56 (38.1)	
Yes	188 (62.7)	97 (63.4)	91 (61.9)	

**Notes:** Values are presented as numbers (percentages). Percentages in the Total column were calculated for the overall sample, whereas percentages in the OMFS and GD columns were calculated within each professional group. Between-group comparisons were performed using Pearson’s chi-square test. OMFS: oral and maxillofacial surgeons; GD: general dentists. * *p* < 0.05 was considered statistically significant.

**Table 2:** Total Knowledge Scores According to Professional Group and Selected Variable.

(**A**) **Comparison of total knowledge scores between professional groups**					
**Professional Group**	**Median (Min–Max, IQR)**		**Z**	***p*-Value**	**Effect Size (r)**
OMFS	41 (26–47, 8)		−6.532	<0.001 *	0.377
GD	36 (13–47, 7)				
(**B**) **Comparison of total knowledge scores according to selected variables**					
**Variable**	**Category**	**n**	**Median (IQR)**	**Test**	***p*** **-Value**
Total knowledge score	Overall	300	39 (9)	—	—
Reliability of coronectomy	Yes	194	39 (9)	Kruskal–Wallis	<0.001 *
	No	31	41 (4)		
	I do not know	75	35 (7)		
Self-reported decision-making confidence: coronectomy vs. extraction	Yes	149	40 (9)	Mann–Whitney U	<0.001 *
	No	151	37 (8)		
Interest in continuing education on coronectomy	Yes	173	39 (9)	Mann–Whitney U	0.244
	No	127	39 (9)		
Preference for 3D imaging	Yes	265	39 (9)	Mann–Whitney U	0.005 *
	No	35	37 (8)		
Experience with coronectomy	None	191	38 (8)	Kruskal–Wallis	<0.001 *
	<5 cases	54	40 (8)		
	≥5 cases	55	42 (9)		
Heard of coronectomy	Yes	280	39 (9)	Mann–Whitney U	0.002 *
	No	20	34.5 (11)		
Professional experience (years)	<5 years	189	39 (8)	Kruskal–Wallis	0.192
	5–10 years	82	38 (9)		
	>10 years	29	44 (12)		
Workplace sector	Public	251	37 (8)	Mann–Whitney U	0.081
	Private	49	39 (9)		

**Notes:** Values in section A are presented as medians (minimum–maximum, interquartile range). Values in section B are presented as medians (interquartile range). The Mann–Whitney U test was used for comparisons between two groups, and the Kruskal–Wallis test was used for comparisons among three or more groups. The effect size in section A was calculated as r = |Z|/√N. IQR: interquartile range; OMFS: oral and maxillofacial surgeons; GD: general dentists. * *p* < 0.05 was considered statistically significant. When significant differences were detected in comparisons involving three or more groups, Bonferroni-adjusted post hoc analyses were performed.

**Table 3:** Comparison of selected coronectomy-related variables between OMFS and GD.

Variable	Response	OMFS n (%)	GD n (%)	Test Statistic	*p*-Value
Reliability of coronectomy	Yes	116 (75.8)	78 (53.1)	χ^2^ = 35.894	<0.001 *
	No	21 (13.7)	10 (6.8)		
	I do not know	16 (10.5)	59 (40.1)		
Self-reported decision-making confidence: coronectomy vs. extraction	No	29 (19.0)	122 (83.0)	χ^2^ = 122.986	<0.001 *
	Yes	124 (81.0)	25 (17.0)		
Interest in continuing education on coronectomy	No	71 (46.4)	56 (38.1)	χ^2^ = 2.121	0.145
	Yes	82 (53.6)	91 (61.9)		
Preference for 3D imaging	No	13 (8.5)	22 (15.0)	χ^2^ = 3.045	0.081
	Yes	140 (91.5)	125 (85.0)		
Experience with coronectomy	None	50 (32.7)	141 (95.9)	χ^2^ = 129.764	<0.001 *
	<5 cases	50 (32.7)	4 (2.7)		
	≥5 cases	53 (34.6)	2 (1.4)		
Heard of coronectomy	No	1 (0.7)	19 (12.9)	χ^2^ = 18.144	<0.001 *
	Yes	152 (99.3)	128 (87.1)		

**Notes**: Values are presented as numbers and percentages within each professional group. χ^2^: chi-square test statistic; OMFS: oral and maxillofacial surgeons; GD: general dentists. * *p* < 0.05 was considered statistically significant.

**Table 4:** Self-perceived knowledge, its correlation with total knowledge score, and comparison between professional groups.

(**A**) **Descriptive statistics of self-perceived knowledge items**				
**Item**	**Mean ± SD**		**Median (Min–Max)**	
Self-perceived knowledge of mandibular third molar surgery complications	3.70 ± 1.02		4 (1–5)	
Self-perceived knowledge of nerve injury	3.40 ± 0.98			
Self-perceived knowledge of coronectomy purpose	3.31 ± 0.96		3 (1–5)	
Self-perceived knowledge of coronectomy complications	3.15 ± 1.00		3 (1–5)	
(**B**) **Correlation between self-perceived knowledge and total knowledge score**				
**Variable**	**ρ**		***p*** **-value**	
Knowledge score vs. self perceived knowledge	0.414		<0.001 *	
(**C**) **Comparison of self-perceived knowledge between groups**				
**Variable**	**Mean ± SD** **(OMFS) (n = 153)**	**Mean ± SD** **(GD) (n = 147)**	**Z**	***p*** **-Value**
Self-perceived knowledge score	3.95 ± 0.65	2.79 ± 0.68	−11.610	<0.001 *

**Notes**: Values in section A are presented as means ± standard deviations and medians (min–max). Spearman’s rank correlation analysis was used in section B, and the Mann–Whitney U test was used in section C. * *p*-value < 0.05 was considered statistically significant. OMFS: oral and maxillofacial surgeons; GD: general dentists; SD: standard deviation; ρ: Spearman’s correlation coefficient; Z: Mann–Whitney U test statistic.

**Table 5:** Self-Reported Complications Following Coronectomy and Knowledge Sources Regarding Coronectomy.

(**A**) **Self-reported complications following coronectomy**	
**Complications**	**n (%)**
Infection	40 (29.6)
Pain	35 (25.9)
Root eruption/migration	30 (22.2)
Swelling/edema	15 (11.1)
Delayed healing	5 (3.7)
Nerve injury	5 (3.7)
Alveolitis	3 (2.2)
Root-related pathological changes	2 (1.5)
(**B**) **Sources of knowledge about coronectomy**	
**Source**	**n (%)**
Undergraduate education	160 (53.3)
Specialty training	55 (18.3)
Scientific literature/textbooks	35 (11.7)
Clinical experience	25 (8.3)
Colleagues/peer learning	15 (5.0)
Courses/seminars/social media	10 (3.3)

**Notes**: In section A, only non-blank responses to Question 34 from participants with previous coronectomy experience were analyzed (52 of 109 participants). Blank responses were treated as missing/non-response and were not interpreted as indicating no complications. Percentages were calculated based on the total number of reported complications (n = 135), as more than one complication could be reported by each participant. In section B, percentages were calculated based on the total number of participants (n = 300).

**Table 6:** Treatment preferences for mandibular third molars in close proximity to the inferior alveolar nerve according to professional group.

Treatment Preference	Response	OMFS n (%)	GD n (%)	Test Statistic	*p*-Value
Extraction	Not selected	46 (30.1)	114 (77.6)	χ^2^ = 67.921	<0.001 *
	Selected	107 (69.9)	33 (22.4)		
Wait-and-see	Not selected	40 (26.1)	25 (17.0)	χ^2^ = 3.688	0.055
	Selected	113 (73.9)	122 (83.0)		
Coronectomy	Not selected	17 (11.1)	50 (34.0)	χ^2^ = 22.671	<0.001 *
	Selected	136 (88.9)	97 (66.0)		
Referral (to specialist)	Not selected	135 (88.2)	44 (29.9)	χ^2^ = 105.896	<0.001 *
	Selected	18 (11.8)	103 (70.1)		
Orthodontic extrusion followed by extraction	Not selected	96 (62.7)	92 (62.6)	χ^2^ = 0.001	0.977
	Selected	57 (37.3)	55 (37.4)		

**Notes**: Values are presented as numbers and percentages within each professional group. Each treatment option was analyzed separately as selected versus not selected because participants could select more than one option. χ^2^: chi-square test statistic; OMFS: oral and maxillofacial surgeons; GD: general dentists. * *p* < 0.05 was considered statistically significant.

**Table 7:** Total knowledge scores according to treatment preferences for mandibular third molars in close proximity to the inferior alveolar nerve.

Treatment Preference	Response	Median (Min–Max, IQR)	Test Statistic	*p*-Value
Extraction	Not selected	37 (13–47, 7.5)	Z = −5.237	<0.001 *
	Selected	41 (25–47, 8)		
Wait-and-see	Not selected	39 (13–47, 10)	Z = −1.182	0.237
	Selected	39 (21–47, 9)		
Coronectomy	Not selected	36 (13–47, 7)	Z = −3.665	<0.001 *
	Selected	40 (21–47, 9)		
Referral (to specialist)	Not selected	40 (25–47, 8)	Z = −5.235	<0.001 *
	Selected	36 (13–47, 8)		
Orthodontic extrusion followed by extraction	Not selected	39 (13–47, 9)	Z = −0.057	0.954
	Selected	39 (24–47, 8)		

**Notes**: Values are presented as medians, interquartile ranges, and minimum–maximum values. Each treatment option was evaluated separately as selected versus not selected because participants could select more than one option. The Mann–Whitney U test was used for comparisons. Z: Mann–Whitney U test statistic; IQR: interquartile range. * *p* < 0.05 was considered statistically significant.

**Table 8:** Multivariable linear regression analysis for factors associated with total knowledge score.

Variable	Reference	B	SE	β	t	*p*-Value	95% CI for B	VIF
Constant	—	38.940	1.980	—	19.670	<0.001 *	35.043 to 42.837	—
GD	OMFS	−3.777	0.612	−0.321	−6.170	<0.001 *	−4.918 to −2.573	1.084
Professional experience: 5–10 years	<5 years	−0.372	0.701	−0.028	−0.530	0.595	−1.752 to 1.008	1.079
Professional experience: >10 years	<5 years	1.012	1.029	0.051	0.980	0.327	−1.013 to 3.037	1.087
Postgraduate continuing education: Yes	No	0.782	0.642	0.064	1.220	0.224	−0.482 to 2.046	1.048
Heard of coronectomy: Yes	No	2.983	1.284	0.126	2.320	0.021 *	0.456 to 5.510	1.071
Preference for 3D imaging: Yes	No	2.639	0.954	0.144	2.770	0.006 *	0.761 to 4.517	1.031

**Model summary:** F = 11.10, *p* < 0.001; R = 0.430; R^2^ = 0.185; adjusted R^2^ = 0.169; Durbin–Watson = 1.639. **Notes: B:** unstandardized regression coefficient; **SE:** standard error; **β:** standardized regression coefficient; **CI:** confidence interval; **VIF:** variance inflation factor; **OMFS:** oral and maxillofacial surgeons; **GD:** general dentists. Reference categories are shown in the table. * *p* < 0.05 was considered statistically significant.

## Data Availability

The datasets supporting the conclusions of this article are not publicly available due to participant confidentiality and ethical restrictions but are available from the corresponding author on reasonable request.

## Supplementary Materials

The following supporting information can be downloaded at: https://www.mdpi.com/article/10.3390/healthcare14172868/s1, File S1: Questionnaire used in the study; File S2: Item-level analysis of the knowledge questions, Table S1. Item-level distribution of correct responses to knowledge questions; File S3: Completed STROBE checklist for cross-sectional studies; File S4: Evidence-based answer key and scoring scheme for the knowledge items. Refs. [5,7,11,30,31,32,33] are cited in Supplementary File S4.

## Abbreviations

The following abbreviations are used in this manuscript:

AAOMS	American Association of Oral and Maxillofacial Surgeons
CVR	Content validity ratio
CVI	Content validity index
GD	General dentists
IAN	Inferior alveolar nerve
IQR	Interquartile range
OMFS	Oral and maxillofacial surgeons
SD	Standard deviation
SPSS	Statistical Package for the Social Sciences
STROBE	Strengthening the Reporting of Observational Studies in Epidemiology
VIF	Variance inflation factor
