# Supplementary material for: Assessment of Knowledge, Clinical Experience, and Clinical Decision-Making Regarding Coronectomy of Mandibular Third Molars Among General Dentists and Oral and Maxillofacial Surgeons: A Cross-Sectional Survey
Source: Healthcare (Basel). 2026 Sep 7;14(17):2868. doi: 10.3390/healthcare14172868 (PMC13566853; doi:10.3390/healthcare14172868)
Supplement: Supplementary file 1 [file healthcare-14-02868-s001.zip › Healthcare_Supplementary_File S1.pdf]

Supplementary File S1

Questionnaire used in the study

Assessment of Knowledge, Clinical Experience, and Clinical Decision-Making Regarding Coronectomy of Mandibular Third Molars Among General Dentists and Oral and Maxillofacial Surgeons: A Cross-Sectional Survey

1. Gender:

□ Male

□ Female

2. Date of birth:

3. How many years of professional experience do you have?

□ <5 years

□ 5–10 years

□ >10 years

4. Where do you work?

□ Private sector

□ Public sector

5. Have you received / are you currently receiving specialty training in oral and maxillofacial surgery?

□ Yes

□ No

6. Have you attended any postgraduate educational activity (course, seminar, symposium, congress, or meeting)?

(If your answer is “No,” you may skip Question 7.)

□ Yes

□ No

7. What was the topic of the educational activity you attended?

……………………………………………………………………..

8. Have you previously performed extraction of an impacted mandibular third molar?

□ Yes

□ No

(Participants who answered “No” to Question 8 may skip Question 9.)

9. How many impacted mandibular third molar extractions have you performed?

□ <10

□ 10–50

□ >50

10. Which of the following are indications for extraction of impacted mandibular third molars?

(You may select more than one option.)

□ Prevention or treatment of pericoronitis

□ Dental caries

□ Orthodontic reasons

□ Teeth associated with odontogenic cysts and tumors

□ Root resorption of the adjacent tooth

□ Prosthetic reasons

□ Prevention of pathological fractures of the jaws

□ Periodontitis

□ Atypical pain

11. Do you think you have sufficient knowledge about possible complications following extraction of impacted mandibular third molars?

□ I have very good knowledge

□ I have good knowledge

□ I have moderate knowledge

□ I do not have sufficient knowledge on this subject

□ I have no knowledge on this subject

12. Which of the following may be encountered as complications after extraction of impacted mandibular third molars?

(You may select more than one option.)

□ Inferior alveolar and lingual nerve injury

□ Alveolar osteitis

□ Bleeding

□ Edema

□ Infection

□ Pain

□ Trismus

13. Do you think you have sufficient knowledge about nerve injuries?

□ I have very good knowledge

□ I have good knowledge

□ I have moderate knowledge

□ I do not have sufficient knowledge on this subject

□ I have no knowledge on this subject

14. Which of the following may cause inferior alveolar nerve injury in the oral and maxillofacial region?

(You may select more than one option.)

□ Local anesthetic injection

□ Third molar surgery

□ Dental implant placement

□ Orthognathic surgery

□ Maxillofacial trauma

□ Endodontic treatment

15. Do you think nerve injury may occur during extraction of an impacted mandibular third molar?

□ Yes

□ No

□ I do not know

16. Which of the following may be considered radiographic markers on panoramic radiography indicating a possible close relationship between the inferior alveolar canal and an impacted mandibular third molar?

(You may select more than one option.)

□ Darkening at the root apex

□ Deviation of the root

□ Narrowing of the root

□ Darkening and bifidity of the root apex

□ Interruption of the radiopaque border of the canal

□ Diversion of the canal

□ Narrowing of the canal

17. What treatment options would you offer for a patient with an impacted mandibular third molar in close relationship with the inferior alveolar nerve?

(You may select more than one option.)

□ Complete extraction

□ Leave and observe

□ Coronectomy

□ Referral to another specialist

□ Orthodontic extrusion followed by extraction

18. Have you heard of coronectomy before?

□ Yes

□ No

19. If yes, please indicate where you heard about it.

……………………………………………………………………………

20. Do you have knowledge about the coronectomy procedure?

□ Yes

□ No

21. In coronectomy, is the root portion left in place while the crown portion is removed?

□ Yes

□ No

□ I do not know

22. Is coronectomy generally performed on impacted mandibular third molars?

□ Yes

□ No

□ I do not know

23. Compared with total extraction, is the incidence of inferior alveolar nerve injury lower with coronectomy?

□ Yes

□ No

□ I do not know

24. Do you think you have sufficient knowledge about the purpose of coronectomy?

□ I have very good knowledge

□ I have good knowledge

□ I have moderate knowledge

□ I do not have sufficient knowledge on this subject

□ I have no knowledge on this subject

25. Which of the following are contraindications for coronectomy?

(You may select more than one option.)

□ Teeth with infection involving the roots

□ Medically compromised patients (e.g., immunosuppressed patients, or those receiving radiotherapy or chemotherapy)

□ Situations in which complete removal of the enamel is not expected to be possible

□ Cases in which the second molars are planned to be orthodontically distalized

□ Cases in which the roots become mobile during coronectomy

□ Caries extending to the roots

□ Horizontal positioning of the tooth along the course of the inferior alveolar nerve

26. Do you think you have sufficient knowledge about complications of coronectomy?

□ I have very good knowledge

□ I have good knowledge

□ I have moderate knowledge

□ I do not have sufficient knowledge on this subject

□ I have no knowledge on this subject

27. Which of the following are complications of coronectomy?

□ Postoperative pain

□ Infection

□ Alveolar osteitis

□ Root migration

□ Root eruption

□ Pathological conditions around the retained roots

□ Nerve injury

□ Bleeding

28. Do you think coronectomy is a reliable method?

□ Yes

□ No

□ I do not know

29. Do you consider yourself competent to decide between coronectomy and total extraction for impacted mandibular third molars?

□ Yes

□ No

30. Would you like to receive training on coronectomy?

□ Yes

□ No

31. Do you prefer three-dimensional imaging methods during the diagnosis of impacted third molars?

□ Yes

□ No

32. Have you ever performed coronectomy before?

□ No

□ <5 cases

□ ≥5 cases

(Participants who answered "No" to Question 32 do not need to answer Questions 33 and 34.)

33. Were you satisfied with the outcomes of the cases in which you performed coronectomy?

□ Yes

□ No

34. What complications did you encounter in the cases in which you performed coronectomy?

……………………………………………………………………………………

THANK YOU FOR PARTICIPATING IN OUR SURVEY.
