# Supplementary material for: Assessment of Knowledge, Clinical Experience, and Clinical Decision-Making Regarding Coronectomy of Mandibular Third Molars Among General Dentists and Oral and Maxillofacial Surgeons: A Cross-Sectional Survey
Source: Healthcare (Basel). 2026 Sep 7;14(17):2868. doi: 10.3390/healthcare14172868 (PMC13566853; doi:10.3390/healthcare14172868)
Supplement: Supplementary file 1 [file healthcare-14-02868-s001.zip › Healthcare_Supplementary_File S2.pdf]

Supplementary File S2

Item-level distribution of correct responses to knowledge questions

Supplementary Table S1. Item-level distribution of correct responses to knowledge questions

Knowledge domain	Item	Correct responses, n (%)
**Indications for impacted mandibular third molar extraction**	Prevention or treatment of pericoronitis	284 (94.7)
	Dental caries	257 (85.7)
	Orthodontic reasons	283 (94.3)
	Teeth associated with odontogenic cysts and tumors	292 (97.3)
	Root resorption of the adjacent tooth	295 (98.3)
	Prosthetic reasons	257 (85.7)
	Prevention of pathological fractures of the jaws	251 (83.7)
	Periodontitis	200 (66.7)
	Atypical pain	229 (76.3)
**Complications after impacted mandibular third molar extraction**	Inferior alveolar and lingual nerve injury	285 (95.0)
	Alveolar osteitis	289 (96.3)
	Bleeding	290 (96.7)
	Edema	287 (95.7)
	Infection	293 (97.7)
	Pain	289 (96.3)
	Trismus	292 (97.3)
**Procedures associated with IAN injury**	Local anesthetic injection	205 (68.3)
	Third molar surgery	299 (99.7)
	Dental implant placement	298 (99.3)
	Orthognathic surgery	297 (99.0)
	Maxillofacial trauma	286 (95.3)
	Endodontic treatment	216 (72.0)
**Radiographic markers indicating possible close relationship with the IAN**	Darkening at the root apex	226 (75.3)
	Deviation of the root	190 (63.3)
	Narrowing of the root	116 (38.7)
	Darkening and bifidity of the root apex	214 (71.3)
	Interruption of the radiopaque border of the canal	280 (93.3)
	Diversion of the canal	205 (68.3)
	Narrowing of the canal	162 (54.0)
**General coronectomy knowledge**	Root retention with crown removal	285 (95.0)
	Usual indication in impacted mandibular third molars	250 (83.3)
	Lower incidence of IAN injury compared with total extraction	281 (93.7)

**Contraindications for coronectomy**	Teeth with infection involving the roots	280 (93.3)
	Medically compromised patients	229 (76.3)
	Inability to completely remove enamel	188 (62.7)
	Planned orthodontic distalization of the second molars	217 (72.3)
	Root mobility during coronectomy	232 (77.3)
	Caries extending to the roots	274 (91.3)
	Horizontal positioning along the course of the IAN	157 (52.3)
**Complications of coronectomy**	Postoperative pain	284 (94.7)
	Infection	277 (92.3)
	Alveolar osteitis	192 (64.0)
	Root migration	215 (71.7)
	Root eruption	204 (68.0)
	Pathological conditions around retained roots	256 (85.3)
	Nerve injury	136 (45.3)
	Bleeding	228 (76.0)

Values are presented as number and percentage of participants who selected the correct response. Percentages were calculated using the total number of participants as the denominator (n = 300). For multiple-response items, each option was evaluated separately. IAN: inferior alveolar nerve.
