# Supplementary material for: Assessment of Knowledge, Clinical Experience, and Clinical Decision-Making Regarding Coronectomy of Mandibular Third Molars Among General Dentists and Oral and Maxillofacial Surgeons: A Cross-Sectional Survey
Source: Healthcare (Basel). 2026 Sep 7;14(17):2868. doi: 10.3390/healthcare14172868 (PMC13566853; doi:10.3390/healthcare14172868)
Supplement: Supplementary file 1 [file healthcare-14-02868-s001.zip › Healthcare_Supplementary_File S3.pdf]

Supplementary File S3

STROBE Statement—Checklist of items that should be included in reports of cross-sectional studies

Reporting guideline used: STROBE checklist for cross-sectional studies.

Section/Topic	Item No.	Recommendation	Reported in manuscript / Location
Title and abstract	1a	Indicate the study's design with a commonly used term in the title or the abstract	Title; Abstract—Methods. The title includes "A Cross-Sectional Study" and the abstract states "cross-sectional questionnaire-based study".
Title and abstract	1b	Provide in the abstract an informative and balanced summary of what was done and what was found	Abstract—Background, Methods, Results, and Conclusions. The abstract summarizes study aim, design/sample, questionnaire, analyses, principal findings, and conclusions.
Introduction	2	Explain the scientific background and rationale for the investigation being reported	Background. The section summarizes the clinical importance of mandibular third molar surgery, IAN injury risk, coronectomy as a nerve-preserving alternative, and inconsistent clinical adoption.
Introduction	3	State specific objectives, including any prespecified hypotheses	Background, final paragraph. The study aim is stated as assessing coronectomy-related knowledge, attitudes, clinical experience, self-reported decision-making confidence, and potential knowledge–practice gaps.
Methods	4	Present key elements of study design early in the paper	Methods, opening paragraph. The manuscript identifies a cross-sectional questionnaire-based study conducted using face-to-face questionnaires and states STROBE reporting.
Methods	5	Describe the setting, locations, and relevant dates, including periods of recruitment, exposure, follow-up, and data collection	Methods; Study Design and Participants. The manuscript reports Turkey as the setting, public/private healthcare institutions and university hospitals as recruitment settings, and June 2024–January 2026 as the data collection period.
Methods	6a	Give the eligibility criteria, and the sources and methods of selection of participants	Methods; Study Design and Participants. Eligibility criteria, professional groups, healthcare settings, convenience sampling, and face-to-face recruitment are described.
Methods	7	Clearly define all outcomes, exposures, predictors, potential confounders, and effect modifiers. Give diagnostic criteria, if applicable	Methods; Study Variables. The primary outcome, main independent variable, secondary variables, and other variables considered in the analyses are defined.
Methods	8	For each variable of interest, give sources of data and details of methods of assessment (measurement). Describe comparability of assessment methods if there is more than one group	Methods; Questionnaire Design and Study Variables. The questionnaire, knowledge scoring, Likert self-perceived knowledge items, open-ended questions, CVR/CVI assessment, and pilot testing are described. The same questionnaire was used for all groups.
Methods	9	Describe any efforts to address potential sources of bias	Methods, opening paragraph; Discussion, limitations. The manuscript states that participation was voluntary, responses were anonymous, and participants were informed that there were no right or wrong consequences. Limitations discuss self-report, recall, response, social desirability, and selection bias.
Methods	10	Explain how the study size was arrived at	Methods; Study Design and Participants. The minimum required sample size was calculated using G*Power with $\alpha = 0.05$ and 80% power; at least 259 participants were required, and 300 were included.
Methods	11	Explain how quantitative variables were handled in the analyses. If applicable, describe which groupings were chosen and why	Methods; Statistical Analysis. Continuous/categorical descriptive statistics are described. Total knowledge score was analyzed as a continuous variable because no validated cut-off values were available.
Methods	12a	Describe all statistical methods, including those used to control for confounding	Methods; Statistical Analysis. The manuscript reports Shapiro–Wilk, Mann–Whitney U, Kruskal–Wallis, Bonferroni-adjusted post hoc tests, Spearman correlation, chi-square tests, qualitative content analysis, and exploratory multivariable linear regression with coefficients, CIs, p-values, and VIFs.
Methods	12b	Describe any methods used to examine subgroups and interactions	Methods; Study Design and Participants; Statistical Analysis. Professional background was recorded for subgroup analyses; comparisons between OMFS and general dentists are reported. Interaction analyses were not performed.
Methods	12c	Explain how missing data were addressed	Methods; Study Design and Participants. Because questionnaires were administered face-to-face, participants were asked to complete all applicable items before submission, and no incomplete questionnaires were included in the dataset.

Methods	12d	If applicable, describe analytical methods taking account of sampling strategy	Methods; Study Design and Participants; Discussion, limitations. Convenience sampling is described and justified. No weighting or sampling-strategy-specific analytical adjustment was applied.
Methods	12e	Describe any sensitivity analyses	Not applicable. No sensitivity analyses were performed.
Results	13a	Report numbers of individuals at each stage of study—eg numbers potentially eligible, examined for eligibility, confirmed eligible, included in the study, completing follow-up, and analysed	Results; Participant Characteristics. A total of 300 completed questionnaires were included in the final analysis, comprising 153 OMFS and 147 general dentists.
Results	13b	Give reasons for non-participation at each stage	Not reported. The manuscript reports that only completed questionnaires were included; non-participation reasons were not systematically recorded.
Results	13c	Consider use of a flow diagram	Not applicable. A flow diagram was not used.
Results	14a	Give characteristics of study participants (eg demographic, clinical, social) and information on exposures and potential confounders	Results; Participant Characteristics; Table 1. Demographic and professional characteristics are reported.
Results	14b	Indicate number of participants with missing data for each variable of interest	Methods; Study Design and Participants; Results; Participant Characteristics. No incomplete questionnaires were included in the dataset; 300 completed questionnaires were analyzed.
Results	15	Report numbers of outcome events or summary measures	Results sections 3.2–3.10; Tables 2–8; Additional file 1. Knowledge scores, self-perceived knowledge, clinical experience, complications, knowledge sources, treatment preferences, and regression results are reported.
Results	16a	Give unadjusted estimates and, if applicable, confounder-adjusted estimates and their precision (eg, 95% confidence interval). Make clear which confounders were adjusted for and why they were included	Results; Tables 2–8. Unadjusted comparisons are reported with p-values, medians/IQRs, and selected 95% CIs. Exploratory regression reports adjusted coefficients, 95% CIs, and variables included in the model.
Results	16b	Report category boundaries when continuous variables were categorized	Methods; Statistical Analysis; Tables. Professional experience categories are reported (<5, 5–10, >10 years). Coronectomy experience categories are reported (none, <5 cases, ≥5 cases). Knowledge score was not categorized and was analyzed continuously.
Results	16c	If relevant, consider translating estimates of relative risk into absolute risk for a meaningful time period	Not applicable. This cross-sectional survey did not estimate relative risks over time.
Results	17	Report other analyses done—eg analyses of subgroups and interactions, and sensitivity analyses	Results sections 3.4–3.10. Item-level analysis, subgroup comparisons, self-perceived knowledge correlation, open-ended response content analysis, treatment preference analyses, and exploratory regression are reported. No sensitivity analyses or interaction analyses were performed.
Discussion	18	Summarise key results with reference to study objectives	Discussion, first paragraph. Key findings are summarized in relation to coronectomy-related knowledge, clinical experience, confidence, treatment preferences, subgroup differences, and the knowledge–practice gap.
Discussion	19	Discuss limitations of the study, taking into account sources of potential bias or imprecision. Discuss both direction and magnitude of any potential bias	Discussion, limitations paragraph. Cross-sectional design, self-report, recall/response/social desirability bias, convenience sampling, limited generalizability, absence of case scenarios/clinical outcomes, and exploratory regression limitations are discussed. Direction of selection bias is noted as possible overrepresentation of accessible or oral-surgery-interested dentists.
Discussion	20	Give a cautious overall interpretation of results considering objectives, limitations, multiplicity of analyses, results from similar studies, and other relevant evidence	Discussion and Conclusions. The manuscript uses cautious language such as "associated with," "may," and "suggest," compares findings with previous survey-based studies, and interprets regression findings as exploratory rather than causal.
Discussion	21	Discuss the generalisability (external validity) of the study results	Discussion, limitations paragraph. The manuscript states that convenience sampling limits generalizability to all dentists in Turkey.
Other information	22	Give the source of funding and the role of the funders for the present study and, if applicable, for the original study on which the present article is based	Declarations; Funding. The manuscript states that the authors received no financial support for the research, authorship, and/or publication of the article.

Notes

- Information should be given separately for exposed and unexposed groups where applicable; in this manuscript, OMFS and general dentists are reported separately in the relevant subgroup tables.

- The checklist is completed using section/table locations rather than page/line numbers. Page and line numbers can be added after the final Word file is line-numbered and locked for submission.

• Before submission, ensure that supplementary file numbering is consistent across the manuscript and uploaded files.
