# Supplementary material for: Assessment of Knowledge, Clinical Experience, and Clinical Decision-Making Regarding Coronectomy of Mandibular Third Molars Among General Dentists and Oral and Maxillofacial Surgeons: A Cross-Sectional Survey
Source: Healthcare (Basel). 2026 Sep 7;14(17):2868. doi: 10.3390/healthcare14172868 (PMC13566853; doi:10.3390/healthcare14172868)
Supplement: Supplementary file 1 [file healthcare-14-02868-s001.zip › Healthcare_Supplementary_File S4.pdf]

Supplementary File S4. Evidence-based answer key and scoring scheme for the knowledge items

For multiple-response knowledge questions, each correct response option selected by the participant was awarded 1 point, whereas each unselected correct response option was awarded 0 points. No negative scoring was applied. For Q21–Q23, “Yes” was scored as 1 point, whereas “No” and “I do not know” were scored as 0 points. The maximum possible total knowledge score was 47.

Question	Scored response option / correct answer	Score	Supporting reference(s)
**Q10**	Prevention or treatment of pericoronitis*	1	[30]
Q10	Dental caries*	1	[30]
Q10	Orthodontic reasons*	1	[30]
Q10	Teeth associated with odontogenic cysts and tumors*	1	[30]
Q10	Root resorption of the adjacent tooth*	1	[30]
Q10	Prosthetic reasons*	1	[30]
Q10	Prevention of pathological fractures of the jaws*	1	[30]
Q10	Periodontitis*	1	[30]
Q10	Atypical pain*	1	[30]
**Q12**	Inferior alveolar and lingual nerve injury	1	[31]
Q12	Alveolar osteitis	1	[31]
Q12	Bleeding	1	[31]
Q12	Edema	1	[31]
Q12	Infection	1	[31]
Q12	Pain	1	[31]
Q12	Trismus	1	[31]
**Q14**	Local anesthetic injection	1	[32]
Q14	Third molar surgery	1	[32]
Q14	Dental implant placement	1	[32]
Q14	Orthognathic surgery	1	[32]
Q14	Maxillofacial trauma	1	[32]
Q14	Endodontic treatment	1	[32]
**Q16**	Darkening at the root apex	1	[33]
Q16	Deviation of the root	1	[33]
Q16	Narrowing of the root	1	[33]
Q16	Darkening and bifidity of the root apex	1	[33]
Q16	Interruption of the radiopaque border of the canal	1	[33]
Q16	Diversion of the canal	1	[33]
Q16	Narrowing of the canal	1	[33]
**Q21**	Yes — the crown is removed while the root portion is intentionally retained	1	[5]
**Q22**	Yes — coronectomy is generally performed for impacted mandibular third molars	1	[5,7]
**Q23**	Yes — inferior alveolar nerve (IAN) injury is less frequent with coronectomy than with total extraction	1	[5]
**Q25**	Teeth with infection involving the roots	1	[7]

Q25	Medically compromised patients (e.g., immunosuppressed patients, or those receiving radiotherapy or chemotherapy)**	1	[5]
Q25	Situations in which complete removal of the enamel is not expected to be possible	1	[7]
Q25	Cases in which the second molars are planned to be orthodontically distalized	1	[7]
Q25	Cases in which the roots become mobile during coronectomy	1	[7]
Q25	Caries extending to the roots	1	[7]
Q25	Horizontal positioning of the tooth along the course of the inferior alveolar nerve	1	[7]
**Q27**	Postoperative pain	1	[5,11]
Q27	Infection	1	[5,11]
Q27	Alveolar osteitis	1	[11]
Q27	Root migration***	1	[7,11]
Q27	Root eruption	1	[11]
Q27	Pathological conditions around the retained roots	1	[5,11]
Q27	Nerve injury	1	[5,11]
Q27	Bleeding	1	[11]
	**Maximum total score**	**47**	

* The response options in Q10 represent potential indications for mandibular third molar extraction and should be interpreted in the appropriate clinical context. They should not be construed as universal indications for routine prophylactic removal of asymptomatic, disease-free third molars. Clinical decision-making should consider the presence and severity of symptoms or disease, the feasibility of alternative treatment, individual risk–benefit assessment, and planned dental or surgical treatment.

** This category refers to medical conditions associated with an increased risk of local infection or impaired healing, including immunosuppression, concurrent chemotherapy, or a history of radiotherapy to the mandible, rather than to all medically compromised patients.

*** Root migration is a frequently reported procedure-related outcome following coronectomy and is not necessarily an adverse event per se; however, it was included and scored as a coronectomy-related complication in the original questionnaire.
